# Integrating trauma- and violence-informed care for adolescent mothers in Rwanda: a qualitative study with community health workers

**DOI:** 10.1186/s12913-024-11352-x

**Published:** 2024-07-30

**Authors:** Aimable Nkurunziza, Victoria L. Smye, C. Nadine Wathen, Kimberley T. Jackson, David F. Cechetto, Panagiota Tryphonopoulos, Darius Gishoma

**Affiliations:** 1 School of Nursing and Midwifery, College of Medicine and Health Sciences, University of Rwanda, Kigali, Rwanda; 2 Arthur Labatt Family School of Nursing, Faculty of Health Sciences, Western University, London, ON Canada; 3 Lawrence Bloomberg Faculty of Nursing, University of Toronto, Toronto, ON Canada; 4 Schulich School of Medicine & Dentistry, Western University, London, ON Canada; 5 Mental Health Division, Rwanda Biomedical Center, Ministry of Health, Kigali, Rwanda; 6 School of Nursing, Nipissing University, North Bay, Canada

**Keywords:** Community Health workers, Adolescent mothers, Community, Trauma- and violence-informed care, Universal Health Coverage, Low- and middle-income countries

## Abstract

**Introduction:**

In Rwanda, maternal community health workers play a critical role to improving maternal, newborn and child health, but little is known about their specific experiences with adolescent mothers, who face unique challenges, including trauma, ongoing violence, stigma, ostracism, mental health issues, barriers within the healthcare system, and lack of access to the social determinants of health. This study explored the experiences of maternal community health workers when caring for adolescent mothers in Rwanda to inform the delivery of trauma- and violence-informed care in community maternal services.

**Methods:**

Interpretive Description methodology was used to understand the experiences of 12 community health workers purposively recruited for interviews due to their management roles. To gain additional insights about the context, seven key informants were also interviewed.

**Findings:**

Maternal community health workers provided personalized support to adolescent mothers through the provision of continuity of care, acting as a liaison, engaging relationally and tailoring home visits. They reported feeling passionate about their work, supporting each other, and receiving support from their leaders as facilitators in caring for adolescent mothers. Challenges in their work included handling disclosures of violence, dealing with adolescent mothers’ financial constraints, difficulties accessing these young mothers, and transportation issues. Adolescent mothers’ circumstances are generally difficult, leading to self-reports of vicarious trauma among this sample of workers.

**Conclusion:**

Maternal community health workers play a key role in addressing the complex needs of adolescent mothers in Rwanda. However, they face individual and structural challenges highlighting the complexities of their work. To sustain and enhance their roles, it is imperative for government and other stakeholders to invest in resources, mentorship, and support. Additionally, training in equity-oriented approaches, particularly trauma- and violence-informed care, is essential to ensure safe and effective care for adolescent mothers and to mitigate vicarious trauma among maternal community health workers.

**Supplementary Information:**

The online version contains supplementary material available at 10.1186/s12913-024-11352-x.

## Introduction

Maternal mortality and morbidity continue to be a major concern in developing counties. As of 2020, according to the World Health Organization [1], low- and middle-income countries (LMICs) accounted for 95% of maternal deaths worldwide. “A woman’s lifetime risk of maternal death is the probability that a 15-year-old woman will eventually die from a maternal cause” which translates to one in 5300 in high-income countries compared to one in 49 in low-income countries [1]. Data from 144 countries and territories showed that maternal mortality risk is increased among adolescents aged 15–19 compared with older women [2]. Adolescent girls aged 15–19 years have a two-fold higher risk of dying in childbirth than women aged 20 years and above [3]. Globally, there were 42.5 adolescent births per 1000 women in 2021, down from 64.5 in 2000. Even though there is a remarkable decline in other parts of the world, there were slower declines in sub-Saharan Africa (SSA) regions, with 53.2 births per 1000 women in 2021 [4]. In some areas of the world, maternal mortality is high due to inequalities in access to quality healthcare [5].

Barriers for adolescent mothers to access and utilize maternal services in LMICs include transportation, cultural beliefs, lack of family support, economic factors, and quality of care [6]. A systematic scoping review found that many adolescents in SSA countries don’t access and utilize maternity services during pregnancy; several individual, interpersonal, organizational, and systemic factors contribute to low access and utilization. Individual factors included level of education, age, residential area, socio-economic status, and knowledge of/perceived need for maternal healthcare. Interpersonal or family factors include family traditions, partner’s knowledge, education and perceptions, peer support and the availability of family members’ support. Organizational factors include availability and accessibility of services, and healthcare providers’ collective attitudes towards adolescent pregnancy. Systemic factors include poverty, cultural and religious beliefs, traditional practices, and social stigma [7]. Other studies demonstrated that it is difficult for adolescent mothers to access health services, including lacking the financial support to do so. In addition, lack of privacy and being discriminated against and disrespected by health workers keep young women from care [6, 8]. For example, a South African study found that adolescent mothers often fear others’ reactions because the family, community and those working in health facilities believe adolescent pregnancy to be deviant, shameful, and irresponsible, impacting how they access and utilize maternal services [9]. Consequently, adolescent mothers may avoid available services, meaning that community health workers (CHWs) play a vital role in liaising between adolescent mothers and health services.

CHW programs play a critical role in achieving Universal Health Coverage (UHC) and other global goals, including the Sustainable Development Goals (SDGs) related to health [10]. CHW programs have been implemented to alleviate the shortage of healthcare workers in formal healthcare systems and promote access to and utilization of healthcare services at a community level [11]. A CHW is an individual who has no formal medical training and has been elected by other citizens in the village where they reside to provide basic health services [12]. CHWs are well positioned geographically, culturally and socially to improve maternal and child health in LMIC communities [12–15].

Currently, Rwanda has 45,516 CHWs and the services they offer include prevention, screening and treatment of malnutrition, integrated management of childhood illnesses, family planning, maternal-newborn health, treatment of HIV, TB and other infectious diseases, and behavior change communication [16]. Three CHWs are assigned to each Rwandan village, with one female who delivers maternal and child health interventions (maternal community health workers [MCHWs])- commonly known as Agents de Santé Maternelle (ASMs) and two male-female pairs called Binômes. MCHWs provide maternal and newborn care at home and in follow-up visits; one MCHW is responsible for one village, or approximately 100–150 households [17]. Despite the challenges faced by MCHWs in Rwanda, such as supply shortages, transportation, lack of formal places to use for patient care, low pay and other financial constraints, many are passionate about their work [18]. As part of a functioning system, MCHWs play a crucial role in strengthening primary healthcare and improving the health of underserved populations [10]. In the face of health inequities in LMICs, CHW programs have effectively reached disadvantaged groups, including adolescent mothers, by extending access to healthcare services [19].

Studies of the role of MCHWs in maternal-child health in Rwanda have found that MCHWs promote access to health services, both through outreach and drop-in services [20, 21]; that MCHWs save the lives of women with severe conditions by getting them to acute care facilities via ambulance or other transfer, or sometimes by themselves taking them to hospital [20, 22]. However, mothers in the community reported that sometimes they were not satisfied with services provided by MCHWs, for example, there was not enough information provided, insufficient emotional support and gaps in helping them solve practical problems [23]. These mismatches in service needs and provision could stem from a range of factors, such as mothers’ paths to pregnancy, exposure to trauma, the impact of ongoing violence, including structural and gender-based violence, and the lack of social determinants of health for both MCHWs and mothers [24]. These mismatches, and their effects, highlight the need for improved training and support for MCHWs to understand their own, and mothers’, conditions and experiences; we propose trauma- and violence-informed care (TVIC) as a guiding framework for such education and support.

TVIC is increasingly recognized as a widely applicable and highly relevant universal approach to providing care across various settings [25]. TVIC strives to create a safe environment for people using services by addressing trauma and its relationship to health and behavior, as well as highlighting the interplay between systemic and interpersonal violence and structural inequality [25]. In order to operate, TVIC must follow four principles: (1) understand trauma and violence, including structural and interpersonal violence, and their effects on someone’s life and behavior; (2) provide clients and service providers with a safe and supportive environment emotionally, culturally and physically; (3) provide clients with opportunities for choice, collaboration, and connection; and (4) support clients based on their strengths and through capacity building [25]. These principles are flexible and highly tailored to meet the needs of different practice contexts and organizations; each must be applied at the individual level (i.e., MCHWs interactions with adolescent mothers) and, most importantly, at the organizational level, which establishes the conditions for practice through culture, policies and protocols.

In Rwanda, the latest Demographic Health Survey showed that 5% of teenage girls were either pregnant or already mothers. The Eastern province has the highest rate, with 6.4%, surpassing the national average [26]. A rapid survey by the Collectif des Ligues et Associations de Défense des Droits de l’Homme au Rwanda (CLADHO) across ten districts reported that three-quarters of adolescent pregnancies in Rwanda were linked to sexual violence [27]. Due to socio-cultural factors, some adolescent mothers reported facing social stigma, rejection from their families and communities, and isolation [28], leading to poor mental health outcomes [29], including trauma. Given this context, providers supporting these adolescent mothers must recognize that their trauma history can influence how they seek and use care [30]. If systems and providers do not adopt TVIC principles, there is a risk of both re-traumatization and a lost opportunity to provide support aligned with their health and social needs [25]. However, the literature on how MCHWs connect with adolescent mothers in Rwanda is scarce. Therefore, this study explored the experiences of MCHWs when working with adolescent mothers in Rwanda to inform the delivery of TVIC in community maternal services.

## Methods

### Study design

We used a descriptive, cross-sectional design and Interpretive Description (ID) qualitative methodology [31], following the guidelines specified in the Consolidated Criteria for Reporting Qualitative Research (COREQ), to explore the experiences of MCWHs when working with adolescent mothers. ID’s explicit action orientation means it’s well-suited to studies that want to influence practice [32], a key goal of this research.

### Setting

Eight communities in the selected district of the Eastern province of Rwanda provided the study sites, with data collected from December 2021 to March, 2022. We recruited study participants from the communities in the catchment areas of eight health centres. This area was selected because it has the highest teenage pregnancy rate among Rwandan provinces [33].

### Study population

We recruited MCHWs because of their roles of identifying pregnant women, following up with pregnant women and their newborns and encouraging utilization of antenatal care [34]. Purposive sampling was used to select the MCHWs who met inclusion criteria: being in charge of maternal and child health, having worked with adolescent mothers for at least one year in the community and being willing to share their experiences. To gain in-depth insights about MCHWs experiences, seven key informants (KIs) (four managers of the health centers and three community health officers [CHOs]) were also interviewed as they oversee the work of MCHWs.

### Data collection procedure

Ethical approvals (see below) and the permission letter from the district were presented to the managers of the eight health centers to request access to MCHWs, who are supervised by the health center office and the CHO. The latter helped us to know when the MCHWs’ meetings were scheduled so that we could meet them. We presented our study in their monthly meeting and gave them the letter of information, which we reviewed with them, including answering questions. We asked potential participants within a week to schedule an appointment. Those who agreed to participate and met the inclusion criteria, signed the informed consent forms and interviews were conducted in a private place (offices in the health centres). Due to the nature of the topic and since all MCHWs are women, we recruited two experienced female research assistants (RAs), familiar with the local culture and language, to conduct the interviews. The RAs were trained by the lead researcher (AN) on the study, TVIC, ID methodology and exercising reflexivity. AN interviewed KIs. Regular debriefing meetings between AN and the RAs were conducted after each interview to discuss observations, clarify any uncertainties, and ensure the accuracy and completeness of the collected data.

Semi-structured interview guides developed by the research team in Kinyarwanda (the primary language spoken by the majority of the population) were used (Supplementary file 1&2), including these sample questions (translated to English):

1. *As you know*,* not all*,* but many adolescent mothers have experienced sexual violence. How do you ensure adolescent mothers experience safety and comfort in the home visit?*
2. *Tell me about the philosophy of care provision as it pertains to adolescent mothers and home visiting within perinatal services.*
3. *What are the highlights (and challenges) of your work with adolescent mothers?*
4. *Can you tell me about your overall experience caring for adolescent mothers? Perhaps you could provide a couple of examples of what care you have provided. For example*,* can you tell me a story about caring for an adolescent mother in the home visit that you felt went well? and/or a story about caring for an adolescent mother in the home visit that you felt did not go well? What did you do to handle the situation?*

Interviews were recorded with consent, and two study participants asked for the recorder to be paused for some questions; key comments made while the recorder was off, were noted by the researcher with consent from the participant. These comments generally elaborated on the support received from different stakeholders.

We stopped conducting interviews based on the information power method, which offers a more logical and defensible approach compared to data saturation [35]. Information power posits that a smaller number of participants is sufficient when recruitment seeks participants who hold the richest and most relevant information to answer the research question [36]. Because we recruited experienced participants who were interviewed by experienced RAs, 12 MCHWs along with seven KIs were sufficient to yield rich data. Field notes were taken during and after each interview and incorporated into data analysis. The shortest interview was 31 min while the longest was 73 min. After the interview, each study participant received an honorarium of 8,000 Rwandan francs to compensate for their time, meal, transport, and cell phone costs.

### Data analysis

Data analysis was an iterative process, conducted concurrently with data collection. The lead researcher (AN), fluent in both Kinyarwanda and English, transcribed the data verbatim, and translated it back into English. Field notes were kept separate but linked with interview data after transcription. After cleaning, transcripts were imported to Dedoose software for coding. Data were thematically analyzed using six steps including data familiarization, coding, generating themes, reviewing themes, defining and naming themes, and reporting [37]. Researchers AN and VS read and reread a set of data, then the coding process began. The coders organized regular meetings, engaging in open dialogue to address and settle coding discrepancies. Their objective was to achieve consensus through collaborative discussions. A list of codes was grouped into categories and presented to the whole team, allowing them to identify themes by looking for patterns. The development and exploration of these themes remained provisional as we progressed with our analysis during the meetings, and as interviews progressed. By adopting this approach, we could modify or restructure the themes as needed, ensuring they could be adapted, and/or redefined at either higher or lower levels of abstraction, allowing for enhanced flexibility and adaptability in our research [38].

### Rigor

To uphold the integrity and rigor of our findings, we meticulously adhered to the four evaluative criteria for interpretive description (ID) studies as delineated by Thorne [39]: epistemological integrity, representative credibility, analytic logic, and interpretive authority. To satisfy these criteria, we implemented various strategies, including acknowledging the epistemological stance congruent with our research question, identifying logically consistent interpretive strategies, engaging in reflexive journaling, debriefing meetings, employing methodological triangulation, maintaining an audit trail, scrutinizing field notes and member checking.

### Ethics

Ethical approval was obtained from Western University Health Science Ethics Board (HSREB, project ID: 119,846) and the University of Rwanda, College of Medicine and Health Sciences Institutional Review Board (Approval Notice: No 330/CMHS IRB/2021). After being informed about the secure handling of their data and assured of their anonymity, all participants provided written informed consent. Participation in the study was entirely voluntary, and participants were assured that neither their decisions regarding participation, nor anything they said, would affect their work. Personal data was anonymized during transcription, and no identifiable details were included in reporting findings.

## Results

The basic demographic characteristics of the 12 MCHW interview participants are shown in Table 1 and of the seven KI interview participants in Table 2.

**Table 1:** Socio-demographic characteristics of MCHWs

Pseudonym	Age	Gender	Level of education	Experience as MCHW
MCHW-A	48	Female	Senior 3*	3 years
MCHW-B	44	Female	Senior 2	2 years
MCHW-C	56	Female	Senior 3	5 years
MCHW-D	43	Female	Senior 2	4 years
MCHW-E	42	Female	Primary 6	4 years
MCHW-F	41	Female	Primary 5	2 years
MCHW-G	63	Female	Senior 3	12 years
MCHW-H	54	Female	Senior 3	5 years
MCHW-I	38	Female	Primary 6	9 years
MCHW-J	43	Female	Senior 1	8 years
MCHW-K	50	Female	Senior 3	11 years
MCHW-L	46	Female	Senior 2	3 years

*Senior 3 high school = grade 9

**Table 2:** Socio-demographic characteristics of KIs

No	Title	Age	Gender	Level of education	Working experience
KI-1	Head of the health center	45	Male	A1	15 years
KI-2	Community health officer	34	Male	A0	7 years
KI-3	Community health officer	36	Male	A0	6 years
KI-4	Head of health center	48	Female	A0	17 years
KI-5	Head of health center	42	Male	A1	1 year
KI-6	Community health officer	30	Female	A0	1 year
KI-7	Head of health center	36	Male	A1	1 year

### Themes

Our analysis of MCHWs’ experiences working with adolescent mothers identified four conceptual themes : (a) personalized support for adolescent mothers; (b) facilitators to connect with adolescent mothers; (c) contextual challenges; and (d) vicarious trauma (Table 3).

**Table 3:** Major themes and sub-themes arising from codes

Selected codes	Sub-themes	Themes
Follow-up/frequent visits	Continuity of care	Personalized support for adolescent mothers
Community-based support		
Monitoring adherence to the health centers’ appointments		
Support and guidance		
Building trust in healthcare services	A MCHW as a liaison	
Encouraging health facility visits		
Promoting family acceptance		
Family mediation		
Parental guidance		
Advocacy		
Collaboration with local authorities		
Protective role		
Active listening	Relational engagement	
Positive relationships		
Creating a safe space for expression		
Frequent visits	Tailored home visits	
Gradual approach		
Patience		
Encourage openness		
Understanding family dynamics		
Passion for work	Love of the job	Facilitators to connect with adolescent mothers
Devotion and commitment		
Future-oriented		
Peer learning	Peer support and collegiality	
Knowledge sharing		
Support network		
Training	Structural support	
Supervision		
Emergency response		
Trust issues	Handling disclosure	Contextual challenges
Lack of response		
Referral		
Financial assistance	Dealing with adolescent mothers’ financial constraints	
Emergency support		
Provision of basic needs		
Difficulty in locating adolescent mothers	Difficulties in accessing adolescent mothers	
Long distance challenges		
Parental interference and hiding		
Knowledge gaps	Inadequate training	
Training attendance variability		
Need for training		
Emotional responses during interactions	Impact of adolescent mothers’ stories on MCHWs’ daily activities	Vicarious trauma
Personal identification and connection		
Impact on personal life and well being		
Need for psychological support		

### Theme 1: personalized support for adolescent mothers

Modifying reproductive health behaviors is one of the strategies used to provide personalized support to adolescent mothers in their communities. These strategies include ensuring continuity of care, acting as liaison (in perinatal services, family, and local services), developing a MCHW-adolescent positive relationship through relational engagement, and tailoring home visits to the unique needs of the adolescent.

#### Continuity of care

The normative roles of MCHWs overseeing the care of mothers and their newborns in Rwanda include providing specific services to pregnant women and new mothers and their infants up to 2 months old. An example of this would be antenatal visits. In their local communities, MCHWs identify, register, and refer pregnant women to health centers for antenatal care. Given the unique needs of adolescent mothers, MCHWs play an important role in care provision from the time they suspect that the adolescent mother is pregnant to delivery, and on to when her baby is approximately 2 months old – referred to as provision of “continuity of care.” As one MCHW said,

*My role would be to promote the adolescent mother’s health from the time I realize she is pregnant. When the health center confirms it*,* I monitor her to see if she abides by what the healthcare providers told her*,* for example*,* appointments for antenatal care*,* eating healthy and any other health topic important to a pregnant mom. After birth*,* I ensure that she is breastfeeding well. I monitor this by measuring the baby’s growth until the baby is grown-up. I also encourage vaccination.* (MCHW-G)

MCHWs primarily provide this kind of care to support the ongoing health of adolescent mothers and their babies. As MCHW-D elaborates further below,

*I often visit pregnant adolescent patients throughout their pregnancy as well as after they have given birth. I go to their places since we do not want them to feel uncomfortable in the community. I ensure that I follow her up from day one until she no longer needs the health center’s appointments. My role is to ensure that all appointments are followed up*,* and in case she has a problem; I would immediately take her to the health center or call an ambulance.*

These MCHWs expressed a desire to provide continuity of care and build trust with the adolescent mother to support safe care. According to the MCHWs, due to stigma associated with adolescent pregnancies, adolescent mothers have developed strategies such as avoiding walking through the community to see the MCHWs so they are not seen as accessing perinatal services. MCHWs accommodate the adolescent mothers needs by travelling to the adolescent mother, i.e., they tailor their practice to the unique features of caring for adolescent mothers. This practice reflects the MCHW’s understanding of the impact of stigma and associated trauma and potential violence.

#### MCHWs as liaisons

Some MCHWs promote safe motherhood by acting as the liaison between perinatal services and the adolescent to ensure that adolescent mothers get health services as soon as they are identified in the community. For example, as MCHW-g reported, *“My work is about identifying them [adolescent mothers] in the community so that I can advise them to go to the health facility.”* Another noted, “*Our role is to advise these adolescents and let them know that the healthcare providers will help instead of hiding in the community. We show them that the health center is the safest place compared to anywhere they are.”* (MCHW-F) Identifying adolescent mothers in the community and encouraging them to go to the health facility was felt by MCHWs to increase their access to perinatal services as early as possible, i.e., at the right time.

Furthermore, when adolescents become pregnant, it may create conflicts in families and depending on the family members’ level of understanding, the adolescent may be abused or even abandoned. As a study participant said, *“Teenagers are sometimes abused by their families because they do not understand and accept what has happened to them. I do not only deal with the adolescent mother; I also work with the family.”* (MCHW-D) Another participant further explains their role in the following,

*What we do for them is mainly to reconcile them with their families because after they have gotten pregnant*,* their families tend to blame and reject them for what happened to them. So*,* we approach their families to teach and advise them. We tell them that she is a child like any other and that what happened to them can happen to anyone else. I tell them that rejecting her would affect her and the unborn baby. I start to encourage them to support her in this journey.* (MCHW-J)

Here the MCHW is acting as a liaison between the adolescent mother and her family; they are working to support the provision of an emotionally and physically safe place for the adolescent mother and her unborn child to minimize the potential for abuse or perhaps prevent further abuse of the adolescent mother and promote the overall health and well-being of the adolescent and their baby.

Some MCHWs reported that sometimes they link the adolescent mothers and the family with the local authorities for further investigations, for example, in the case of rape or other abuse. One study participant highlighted the following, *“I work closely with the local authorities because some of those adolescents had been abused*,* especially sharing information with them [local authorities] so they can do something about it.”* (MCHW-K) Another study participant shares this experience:

*When I got there*,* it was apparent that she [adolescent mother] was pregnant*,* but I kept trying to let them admit it on their own without success. So I went to the village authority to seek help because I was afraid of the consequences that it might lead to as her mother and the adolescent kept denying the pregnancy.* (MCHW-B)

In this case the adolescent mother and her mother had refused to go to the health facility regardless of the physical signs of anemia related to pregnancy. In both cases, the MCHWs involved local authorities because they were worried about the safety of the adolescent mother.

A few MCHWs also reported that they advocate for adolescent mothers to the local authorities to support them since most of them struggle to satisfy their basic needs. As one said, *“Some [adolescent mothers] don’t have health insurance*,* but we keep trying to advocate for them to the different level authorities so they can support them.”* (MCHW-B) On one hand, liaising with local authorities and advocating for adolescent mothers was seen as a necessary role to protect and support adolescent mothers and their babies. On the other hand, it could also be a way in which the MCHWs protect themselves from accusations of not doing their duty. However, adolescent mothers face multiple inequities that act as barriers to accessing healthcare services – a reality that MCHWs need to troubleshoot.

#### Relational engagement/inquiry

Engaging with the adolescent mother to build rapport and trust was another important element of care reported by many MCHWs. For example, the study participants said,

*I must behave or possess parental values; otherwise*,* she [adolescent mother] won’t share anything with me. So as a parent*,* I have a responsibility to know and try to address any issues she is struggling with. For example*,* some are new in the area after relocation and searching for a home; others might even be hungry; I have to help them.* (MCHW-A)

*When you visit them [adolescent mothers]*,* you give them space to let their feelings flow*,* like when they want to cry*,* you let them do it*,* but after*,* you approach to comfort them. You try to counsel them slowly and listen to them. We help them understand that life continues after what happened to them; we use real-life examples of people who went into the same situation and how they overcame it.* (MCHW-D)

Relational engagement entails getting to know the client and the contextual features of her life (e.g., financial constraints and other social determinants) so the MCHWs can assist adolescent mothers to address their basic needs; they often provide counselling to foster connection and trust.

#### Tailored home visits

The majority of MCHWs reported that visiting adolescent mothers takes time and needs to be an ongoing process for them to talk and/or admit that they are pregnant. As one participant noted, *“I approach her [adolescent mother] today*,* and it fails*,* I leave*,* but I will come back the next day. Our task is to stick with her until she opens up and tells you what she couldn’t tell*,* even her mother.”* (MCHW-F) As another MCHW said, *“It is not easy; they want to keep information private. They give us a hard time when we first visit. For example*,* admitting that they are at least pregnant is not easy. That’s why I need to go slowly until they accept to be approached.* (MCHW-C)

It took time to engage relationally and build rapport and trust with many of the adolescent mothers. The MCHW supervisors echoed this sentiment. As one supervisor noted, *“We know that our MCWHs are working hard to meet the needs of adolescent mothers. They spend too much time and energy trying to get them to the health center.”* (KI-3) MCHWs structured their work according to the adolescent mothers’ unique needs; they “tailored home visits” and structured their time accordingly. As another MCHW noted,

*There’s an adolescent mother; she’s 18 years old. We are still determining who her father and mother are. It is believed [they] have re-married somewhere else. So she lives with her poor old grandmother*,* who is around 80 years old. This makes life very hard; they need food and clothes. With the current situation of this girl*,* the older woman’s sons find it hard to support all of them*,* which may affect the girl’s behaviours. She’s the most-stubborn in my care. I hardly see her home when I visit*,* most of the case I leave a message to her grandmother to tell her that I miss her because she mostly gets home late in the evening or late at night or she doesn’t come home at all. Sometimes I go there late evening to see if I can meet her. Luckily sometimes I find her there. It takes work.* (MCHW-A)

Adolescent mothers in Rwanda often live in precarious circumstances related to material and social circumstances. The role of the MCHW is shaped by the need to provide care that is responsive to the unique needs of the adolescent during this time. These volunteers often went out of their way to support these young mothers.

### Theme 2: facilitators to connect with adolescent mothers

Despite working in complex situations and environments, the MCHWs reported numerous facilitators that help them work with adolescent mothers, including being passionate about their roles and engaging peer and structural supports.

#### Love of the job

MCHWs are volunteers and reported that they love their job, which motivated them to keep working with adolescent mothers in the community. One study participant highlighted,

*We are volunteers and don’t work for salaries or wages; we do everything for the love of our country only. As we hope our duties have a positive impact on the community. So we try our best to help the community whenever we can and anytime. The only motivation is the outcome of my work. We feel proud to help adolescent mothers*,* and it makes us happy.* (MCHW-A)

In a similar vein, another study participant said the following,

*This work is the work we are passionate about. We don’t regret it. We can leave other jobs we were doing for this. We are devoted to helping them [adolescent mothers] because we know our work will result in a bright future for them. I love this job.* (MCHW-I)

MCHWs showed interest in working with adolescent mothers despite the challenges associated with their role – they prioritized the adolescent mothers’ emotional and physical safety and were led with the hope that this work would make a difference.

#### Peer support and collegiality

To exercise their roles of caring for adolescent mothers, a role they considered demanding, MCHWs recognized the support from their colleagues. As one study participant said, *“I learn from my colleagues on many cases they face*,* especially for adolescent mothers. During our discussion*,* you realize that they face interesting cases*,* and I may share my experience too.”* (MCHW-H) In a similar context, another study participant shared the following, *“We must collaborate and work together in our villages. We have to put together and share our knowledge and information so we can find durable solutions for the problems that those adolescents face.”* (MCHW-E) MCHWs shared the perspective that by collaborating they could find solutions to address the issues adolescent mothers face: to draw awareness to the detrimental effects of trauma and violence on the lives and wellbeing of adolescent mothers. Because of insufficient training, most of the less experienced MCHWs (with fewer than five years of experience) reported that they learn from their more experienced colleagues.

#### Structural support

Even though MCHWs are volunteers, the government of Rwanda tries to support their work by providing some materials, incentives, continuous support and training. For example, as one said,

*Our healthcare center arranges training if there’s something new to share and learn. They call us and give us training. That is the main foundation we use. They can guide us on how to care for adolescents in our community… [However]when an adolescent is about to give birth*,* that’s something out of my scope of practice because I am not a midwife. I have to call my supervisors and make requests*,* for instance*,* an ambulance or assistance.* (MCHW-A)

This support also was noted by a manager of the health center who said, *“We make sure to assist them [MCHWs] whenever they call us”* (KI-7) as well as a supervisor of MCHWs who added, *“We do regular visits to find out if they met any challenges so we can assist to handle them.”* (KI-2) MCHWs added that the government provides them with some basic materials to be used when assuming their roles and responsibilities. One participant explained, *“Yeah*,* we have some raincoats*,* torches*,* registers*,* weight scale*,* pens… all these help us to do our work.”* (MCHWA). A KI added, “We are aware of the challenges they [MCHWs] face and are volunteers, but we try our best to provide means through their cooperatives and the government intervenes.” (KI-1).

### Theme 3: contextual challenges

Challenges identified by MCHWs included handling disclosure, financial constraints, and difficulties in accessing adolescent mothers.

#### Handling disclosures of violence

Some MCHWs shared how they engage with adolescent mothers to gather information related to pregnancy, particularly for those resulted from forced sex. One participant said, *“I make sure that they [adolescent mothers] talk*,* and sometimes we need to know the perpetrator so that the local authorities arrest him. However*,* some of them do not tell this to either their parents or us.”* (MCHW-D) Another study participant expressed the same concern and added that she does everything possible to get information from the adolescent mother, *“If they do not want to talk*,* I can call the local authorities to help me. No challenge can make us give up on any of them.“*(MCHW-E) In this context, another participant added that she uses a different way of taking the adolescent mother to the health facility when they refuse to talk, *“I asked who raped her so they could arrest him*,* they kept quiet. I insist*,* but still*,* most of them do not talk. So I give up and take them to the health facility.”* (MCHW-C) A few of the MCHWs felt it was important for the adolescent mothers to share who their perpetrators were to the authorities – usually with little success. Although the MCHWs had good intentions, i.e., they wanted the perpetrators to be held accountable for their actions, they were not necessarily attuned to the readiness of the adolescent to disclose. Another study participant added another perspective as reflected in the following quote,

*Many adolescents don’t know the perpetrators’ full names due to different circumstances. For instance*,* they get impregnated by people not residents of their current areas*,* so they will only know probably one name*,* which is hard for them. Even though they have been abused*,* their parents keep harassing them*,* asking for the names of those who did it to them*,* which may upset her.* (MCHW-L)

This MCHW recognizes that asking the adolescent mother her partner’s name is upsetting and embarrassing. In this study, the handling of disclosures was found by all MCHWs to be challenging and there was variability in understanding how to do this.

#### Financial constraints

Since they are volunteers, most MCHWs reported spending their own money to solve adolescent mothers’ problems, such as transportation to services and paying for clothes and food. One study participant explained, *“Sometimes you have to help these adolescent mothers in whatever way you can. I can pay little money for some stuff. When I take her to the health center*,* I can pay for her transport when it’s far.”* (MCHW-E) Other participants shared their stories of what happened to them when caring for adolescent mothers,

*For example*,* sometimes a problem needs money to be solved*,* and you don’t have it at that moment. Let’s say you promised her [adolescent mother] to help during her baby delivery*,* which occurs unexpectedly. This becomes very challenging as sometimes getting the money will take work. Our duties are like those of soldiers; we do whatever we can to help them; you need a means of transport to get her to the hospital.* (MCHWC)

Another participant said,

*She [the adolescent mother] had no clothes. She had neither those basic needs for the mother nor the baby. When we got to the hospital*,* as MCHW*,* we tried to do everything possible to help her*,* we found some clothes and food*,* and we did that for her for three months until she moved to another place we didn’t know.* (MCHW-D)

In this study, MCHWs addressed the social determinants of health which could impact adolescent mothers’ access to and utilization of healthcare services. It’s challenging for MCHWs since they are volunteers and do not earn monthly salaries. However, they collaborate with other MCHWs and staff to find basic resources to help adolescent mothers. As one supervisor explains, *“Well*,* we help MCWHs who inform us about an adolescent mother who needs support.”* (KI-6).

#### Difficulties in accessing adolescent mothers

Most MCHWs mentioned that finding adolescent mothers in their homes or locating them takes a lot of work. One study participant said: *“Sometimes*,* you will need help finding them home or going to the wrong address”* (MCHW-A). In a similar context, she highlighted how they can spend more time looking because they are not available for the scheduled visits,

*Let’s say you have to visit two adolescent mothers today*,* and they live in different locations where you have to travel a long distance in between*,* then you end up finding none of them that day. That is a very big problem*,* but we must go back the next day.* (MCHW-A)

The majority of the study participants added that parents also act as barriers to access adolescent mothers as one MCHW said *“Challenges we face are moments like when parents hide adolescent mothers when you visit them. They even deny that she is there. Most challenges come from parents.”* (MCHW-F). In a similar vein another MCHW said, *“Sometimes*,* parents disrespect us by denying us entering their homes.”* (MCHW-G) A supervisor of MCWHs also noted this phenomenon, *“Hmmm…we still have parents who hide them… They do not want MCHWs to access them*,*”* (KI-1) and another supervisor also spoke to this challenge, *“I have been called several times by MCHWs seeking help for parents who refused them entry into their home. MCHWs have information*,* but they cannot enter.”* (KI-6).

Some MCHWs said that they might travel a long distance to reach an adolescent mother due to the area they cover. As one MCHW said, *“I may need to visit an adolescent mother*,* but she stays far. I need some means of transport to get there. When I do not have them*,* it might delay my visit.”* (MCHW-I). Another study participant shared the same challenge:

*During home visits*,* we carry books*,* sometimes three to four*,* and we have to do a long distance*,* which is tiring as the books are heavy plus the long distance we cover … … sometimes you can get an emergency call to attend to one of them [adolescent mothers] late at night*,* and sometimes it will even be raining and all.* (MCHW-I)

Here the MCHW also notes the unexpected challenges that may arise when conducting home visits. Lack of means of transport can delay the home visits or impede MCHWs from attending emergency calls. Some MCHWs added that these problems most often happen to adolescent mothers without family support.

#### Inadequate training

Some MCHWs reported feeling adequately trained or supported. As one MCHW noted, *“Well*,* my counselling knowledge is limited…Sometimes*,* you face a more challenging case than the ones you knew before or your knowledge. You feel like there is a gap.”* (MCHWK) The same concern also was reflected in the study participants’ recommendations to improve perinatal home visits. As a MCHW highlighted, *“The most needed thing is training to broaden our knowledge to help us handle any situation regarding adolescent mothers we may face*,*”* (MCHWH) and as another MCHW noted *“Since I joined them [MCHWs]*,* I did not receive any training*,* but probably others have attended them.”* (MCHW-F).

Although there was a mixed response to training, all participants acknowledged its importance. Supervisors of the MCHWs recommended having specific training to better care for these adolescent mothers in the community. For example, as one supervisor said, *“I also suggest providing more training to MCHWs on managing the challenging cases of adolescent mothers*,* such as trauma.”* (KI-3) In the context of TVIC, the MCHWs’ narratives reflect that MCHWs are not at a level of knowledge and skills to address, as one example, trauma and violence and their impacts on the lives of adolescent mothers.

### Theme 4: vicarious trauma

Some study participants expressed that sometimes they are emotionally affected by the adolescent mothers’ stories and living conditions. As the following participants noted, “*You know*,* sometimes*,* I get carried away by them [adolescent mothers’ stories]*,* especially when I am alone. I get sad*,* but I try to overcome those thoughts because I can’t be able to help them if I can’t help myself.”* (MCHW-H) The MCHWs explained that they mostly get emotionally drained from adolescent mothers without social support. For example, one participant said,

*To every single case*,* especially those who do not have support from their families. You might find that one is your neighbour or your friend’s daughter. When you are talking to her*,* she may cry*,* and you can cry too when you even do not have something to help her in terms of financial means. It is overwhelming! They are our daughters too…. It is tough to absorb it. You can’t sleep when you imagine that it might be your daughter.* (MCHW-C)

Here MCHWs witnessed how they are affected by the stories of adolescent mothers in their daily lives; responses which reflect a significant risk for the negative effects of vicarious trauma. MCHWs’ supervisors explained that they have many adolescents in the community with trauma and other experiences that can affect their providers. One of the KIs noted, *“Given that MCHWs care for people with trauma histories*,* it is understandable that they may develop trauma during their care for adolescent mothers*,* too. So helping them is something we need to solve.”* KI-5 Here, the KIs recognize that MCHWs are at risk of vicarious trauma and are willing to mitigate the risks.

## Discussion

In this study we explored MCHWs’ experiences caring for adolescent mothers in the community. Four main themes were identified including providing personalized support for adolescent mothers, facilitators for connecting with adolescent mothers, contextual challenges, and MCHW’s experiences of vicarious trauma. The findings from this study will contribute to creating safe community perinatal services for both adolescent mothers and MCHWs.

MCHWs reported different strategies they used to provide personalized support for adolescent mothers in their communities. They reported that their roles included identifying a pregnant adolescent and providing consistent care from the first day to when the baby was two months old. The core role of MCHWs to ensure continuity of care was also identified in other studies in Rwanda [21, 40]. This further reinforces the importance of MCHWs in providing essential health services, including to Rwandan adolescents. In addition to providing health education and support, MCHWs ensure that adolescent mothers are well nourished, receive immunizations, and have a safe delivery; they also provided referrals to support services for antenatal and postnatal care. These findings underscore the crucial role that MCHWs play in supporting adolescent mothers. However, the quality of care they provide may be in question, as they lack, by their own reports, formal education and proper training [21].

From the study participants’ narratives, it was found that the MCHWs act as liaisons between adolescent mothers and perinatal services, family, and local services. Connecting adolescent mothers to services aligns with TVIC principle three, which ensures warm referrals and support to address access inequities [25]. In LMICs, MCHWs track pregnant woman and refer them to health facilities and involve local leaders whenever possible [14, 21, 41, 42]. However, MCHWs should consider the needs of adolescent mothers when involving local authorities to ensure that it’s the right time to do so. This implies that adolescent mothers should be consulted about when and how to involve local authorities since some of them might be at the stage of not disclosing their histories particularly those with trauma histories related to sexual abuse. This is consistent with TVIC principle three which emphasizes emotional and physical safety while fostering choice, agency and connection [25].

In the present study, MCHWs support adolescent mothers by developing a positive relationship through relational engagement. It is well documented that MCHWs increase access to care among community members by forming a positive relationship with households [43]. This practice aligns with TVIC principle two, emphasizing safety and trust and building healthy relationships [25]. Adolescent mothers are vulnerable in various ways and require more support and resources. MCHWs stated that they add extra home visits for adolescent mothers compared to the adult mothers due to their unique needs. This aligns with TVIC’s core premise that programs and services should be flexible enough to accommodate people’s different journeys and be tailored to their needs. This demonstrates an equity approach - fundamental to TVIC – that young mothers who need more, get more [25].

MCHWs identified numerous facilitators that helped them work with adolescent mothers. All reported that they love helping adolescent mothers and feel fulfilled even though they work as volunteers. In other studies, MCHWs stated that making a difference in their clients’ lives is rewarding [18, 44]. Clearly, MCHWs are very passionate about their work and strive to make a positive impact with young mothers, and communities as a whole. This implies that if given more support, MCHWs can be better positioned to fulfill their key roles. A few MCHWs reported that when they faced challenges caring for adolescent mothers, they sought advice from colleagues. They often found that their colleagues had valuable insight into the challenges they faced, which helped them to better manage their caseloads. This was reported mainly by less experienced MCHWs, who learn from more experienced colleagues. Therefore, MCHWs can benefit from their peers’ experiences and knowledge in providing effective care, highlighting the need to set up regular experience-sharing meetings regarding adolescent mothers.

In this study, a few MCHWs pressured adolescent mothers to speak out about their situations related to who got them pregnant (rapist/perpetrator) and how the relationship started. Studies have shown that community health workers have limited knowledge of handling disclosures of violence victims [43, 45]. According to the TVIC principles, in order for individuals to receive respectful and safe care, it should not be necessary for individuals to disclose trauma and ongoing violence to receive care [25], and any reporting should be the informed choice of the survivor. Hence, MCHWs must have adequate training and resources to respond appropriately to adolescent mothers’ disclosures.

While MCWHs are volunteers and do not earn a salary, they face the challenges of dealing with adolescent mothers’ financial constraints such as paying for transport money, providing for basic needs such as food and baby clothes. The same concerns were also reported in other studies in Rwanda [18, 21, 41]. However, this is a tremendous burden for MCHWs. Ultimately, it is understandable that MCWHs face a demanding job without the same resources as professional health workers. This struggle is further compounded by the fact that MCWHs often have to balance their financial obligations while dedicating their time and energy to helping adolescent mothers.

Caring for adolescent mothers wasn’t easy for MCHWs due to parents who deny them to access the adolescent or to required information. In contrast, in another study conducted in South-Africa, it was reported that adolescent mothers are encouraged by their mothers to access perinatal services [9]. This might be related to differing socio-cultural and contextual factors. However, it implies that interventions to support adolescent mothers should, if safe according to TVIC principle 2, involve parents [25]. MCHWs also reported a lack of transportation to visit adolescent mothers, in line with other studies [14, 19, 21, 46]. This highlights the urgent need to prioritize access to transportation for MCHWs. This could include providing MCHWs with vehicles or public transportation reimbursement.

Even though some MCHWs reported that they receive training, others mentioned that they did not receive any specific training to care for adolescent mothers, and others reported that they need more training; generally, they identified gaps in their knowledge and skills to care adolescent mothers. These results are consistent with the findings from the studies which have been conducted in African and Asian countries [14, 47]. In other studies in Rwanda MCWHs reported that the training they receive is irregular and they lack an ongoing mentorship [18, 21, 48]. In their study, Olaniran et al., (2019) found that sometimes the expectations for MCHWs are beyond their scope of practice when providing maternal health services and suggested they be provided more training. Evidence shows that CWHs need support and regular mentorship and supervision to ensure they provide quality home visits [14, 44, 49, 50]. Since MCHWs in Rwanda do not have formal education, it is imperative to provide regular training and mentorship to ensure they provide safe and effective care. The MCHWs training module, published in 2010, does not mention the care of ‘adolescent’ mothers; rather, it discusses the care of pregnant women in general, and the training is targeted at adults [51]. Lack of adequate training among MCHWs makes it challenging to provide safe and respectful care to adolescent mothers, which should be one of the components of meaningful organizational and cultural changes for TVIC [25].

Vicarious trauma was also reported by MCHWs as a challenge when caring for adolescent mothers. Vicarious trauma nursing is defined as “a psychological phenomenon that causes a permanent cognitive shift in the inner experience and world views of nurses after prolonged empathetic engagement with a patient’s trauma” [52]. Some study participants expressed that they are sometimes emotionally affected by empathetic engagement with the adolescent mothers’ stories and living conditions such as lack of social support. Findings from one study revealed that the emotional costs might be also related to lack of resources to help vulnerable people [44]. Working in health and social service settings places people at risk of experiencing trauma due to working with many people who have experienced trauma and violence and stressful working conditions and inadequate resources [25]. MCHWs’ capacity to respond to experiences of trauma and violence may be reduced if they are not supported by the Ministry of Health/healthcare settings to practice in a trauma- and violence-informed way and when we fail to recognize and respond to the harm MCHWs themselves may experience while caring for adolescent mothers considered a difficult task [53].

### Strengths and limitations

This study has strengths and limitations to consider when interpreting its findings. First, to the best of our knowledge, this is the first study that explored how MCHWs connect with adolescent mothers in Rwandan communities and how their practice aligns with TVIC principles. Second, to gain deep insights into MCHWs’ experiences when caring for adolescent mothers, we triangulated data by interviewing their supervisors with whom they share their challenges in the daily activities of supporting adolescent mothers. Third, the study recruited willing and ready participants to share their experiences caring for adolescent mothers which generated rich data. Finally, the generated findings can inform policies, programs, and interventions based on TVIC principles to ensure better care for adolescent mothers in Rwandan communities. However, this study does have limitations. First, it was conducted in one region (Eastern province), limiting the findings’ generalizability. Second, we relied on self-reported data, which may be subject to recall bias. Third, since MCHWs reported their practices of supporting adolescent mothers and KIs discussed their leadership roles in supporting MCHWs, there was a potential for social desirability bias. There might be over-reporting of positive practices/roles and under-reporting of undesirable practices/roles [54]. Therefore, future studies should measure MCHWs’ impact more objectively and expand to other provinces. This could include conducting interviews with adolescent mothers and parents or direct observations.

## Conclusion

MCHWs play an essential role in supporting adolescent mothers in Rwanda by providing personalized support through continuity of care, acting as liaisons (among perinatal services, family, and local services), and tailoring home visits to the unique needs of the adolescent. However, MCHWs encounter both individual and structural barriers that hinder the support they provide to adolescent mothers and expose them to vicarious trauma. There is a pressing need to establish maternal community services that ensure the safety and well-being of both adolescent mothers and MCHWs, especially with regard to vicarious trauma. To achieve this, it is crucial to understand the structural and organizational processes that impact MCHWs and adolescent mothers if we want to design workplace cultures that meet young women’s needs [53]. In addition, TVIC training, supervision and ongoing mentorship from supervisors are needed to support the role of MCHWs. Interventions to support parents of adolescent mothers are highly recommended to facilitate easy and safe access to perinatal services for adolescent mothers. TVIC can be used as a tool to address the underlying causes of challenges to care provision and shift community culture, as part of equity-promoting practices and policies.

### Electronic supplementary material

Below is the link to the electronic supplementary material.

Supplementary Material 1

Supplementary Material 2

## Data Availability

The datasets used and/or analyzed during the current study are available from the corresponding author on reasonable request.
