# Supplementary material for: Integrating trauma- and violence-informed care for adolescent mothers in Rwanda: a qualitative study with community health workers
Source: BMC Health Serv Res. 2024 Jul 30;24:868. doi: 10.1186/s12913-024-11352-x (PMC11289957; doi:10.1186/s12913-024-11352-x)
Supplement: Supplementary file 1 — Supplementary Material 1 [file 12913_2024_11352_MOESM1_ESM.docx]

**Interview guide- Maternal Community Health Workers (MCHWs)**

1. Tell me what brought you to participate in this discussion today?
2. Tell me what a home visit looks like with adolescent mothers. What are the unique features of care for adolescent mothers?
3. As you know, not all, but many adolescent mothers have experienced sexual violence. How do you ensure adolescent mothers experience safety and comfort in the home visit? and/or
4. Tell me about the philosophy of care provision as it pertains to adolescent mothers and home visiting within perinatal services.
5. What are the outcomes you hope for in working with adolescent mothers?
6. What are the highlights of your work with adolescent mothers?
7. What are the strengths related to care provision with adolescent mothers?
8. What are the challenges as you see them in providing home visits to adolescent mothers?
9. Can you tell me about your overall experience caring for adolescent mothers? Perhaps you could provide a couple of examples of what care you have provided. For example, can you tell me a story about caring for an adolescent mother in the home visit that you felt went well? and/or a story about caring for an adolescent mother in the home visit that you felt did not go well? What did you do to handle the situation?
10. What was your educational preparation in the care of people who have a violence history? What do you see as your need in this regard?
11. What are your recommendations moving forward to improve the care of adolescent mothers in perinatal services?
