# Supplementary material for: Integrating trauma- and violence-informed care for adolescent mothers in Rwanda: a qualitative study with community health workers
Source: BMC Health Serv Res. 2024 Jul 30;24:868. doi: 10.1186/s12913-024-11352-x (PMC11289957; doi:10.1186/s12913-024-11352-x)
Supplement: Supplementary file 2 — Supplementary Material 2 [file 12913_2024_11352_MOESM2_ESM.docx]

**Invitational questions (key informants)**

1. Can you tell me what interested you in my research study?
2. Tell me what it is like supervising MCHWs activities related to perinatal services?
3. Please share with me your experiences of how home visit is provided to adolescent mothers in the community by MCHWs?
4. What do you see as facilitative to you in creating an environment supportive of MCHWs to do home visits to adolescent mothers in community?
5. What do you see as barriers to you in creating an environment supportive of MCHWS to do home visits to adolescent mothers’ community?
6. In what ways do you offer support to MCHWs to provide the home visits to adolescent mothers in community?
7. What do you think can be done to improve home visits of adolescent mothers in community?
8. Is there anything else can you share with me that you think is important for this research to know how home visits are offered to adolescent mothers, that we did not cover in this discussion?
